# Modulation of Amygdala Response by Cognitive Conflict in Adolescents with Conduct Problems and Varying Levels of CU Traits

**DOI:** 10.1007/s10802-021-00787-z

**Published:** 2021-03-16

**Authors:** Catherine L. Sebastian, Jean Stafford, Eamon J. McCrory, Arjun Sethi, Stephane A. De Brito, Patricia L. Lockwood, Essi Viding

**Affiliations:** 1 Department of Psychology, Royal Holloway University of London, Egham, UK; 2 Clinical, Educational and Health Psychology, University College London, London, UK; 3 School of Psychology, University of Birmingham, Birmingham, UK; 4 Department of Experimental Psychology, University of Oxford, Oxford, UK

**Keywords:** Conduct problems, Callous-unemotional traits, Reactive aggression, Threat, Cognitive conflict, Amygdala

## Abstract

Adolescents with conduct problems and low callous-unemotional traits are characterised by high levels of reactive aggression. Prior studies suggest that they can have exaggerated neural and behavioural responses to negative emotional stimuli, accompanied by compromised affect regulation and atypical engagement of prefrontal areas during cognitive control. This pattern may in part explain their symptoms. Clarifying how neurocognitive responses to negative emotional stimuli can be modulated in this group has potential translational relevance. We present fMRI data from a cognitive conflict task in which the requirement to visually scan emotional (vs. calm) faces was held constant across low and high levels of cognitive conflict. Participants were 17 adolescent males with conduct problems and low levels of callous-unemotional traits (CP/LCU); 17 adolescents with conduct problems and high levels of callous-unemotional traits (CP/HCU, who typically show blunted reactivity to fear), and 18 typically developing controls (age range 10–16). Control participants showed typical attenuation of amygdala response to fear relative to calm faces under high (relative to low) conflict, replicating previous findings in a healthy adult sample. In contrast, children with CP/LCU showed a reduced (left amygdala) or reversed (right amygdala) attenuation effect under high cognitive conflict conditions. Children with CP/HCU did not differ from controls. Findings suggest atypical modulation of amygdala response as a function of task demands, and raise the possibility that those with CP/LCU are unable to implement typical regulation of amygdala response when cognitive task demands are high.

**Supplementary Information:**

The online version contains supplementary material available at 10.1007/s10802-021-00787-z.

## Introduction

Young people with conduct disorder (CD) and conduct problems (CP) exhibit antisocial behaviour that violates the rights of others. This group is heterogeneous, with evidence suggesting the delineation of two subgroups characterised by either high (CP/HCU) or low (CP/LCU) levels of callous-unemotional (CU) traits (e.g. low guilt and empathy and flattened affect; Frick & Viding, 2009; Frick et al., 2014). Such a distinction has recently been adopted by the DSM-5 through the inclusion of a ‘limited-prosocial emotions’ specifier for CD, with these two subgroups showing distinct profiles in terms of aetiology, behaviour, outcomes and neurocognitive profiles (Vanwoerden et al., 2016; Viding & McCrory, 2018). Notably, the two groups differ in their behavioural and neural responses to affective stimuli. Whereas youth with CP/HCU are characterised by attenuated responses to others’ distress and pain (e.g. Jones et al., 2009; Lockwood et al., 2013; Marsh et al., 2008; Viding et al., 2012), those with CP/LCU tend to show exaggerated neural and behavioural affective responses (Viding et al., 2012; Sebastian et al., 2014).

Researchers have posited that differing affective processing profiles may partially account for the types of conduct problems displayed (e.g. Hwang et al., 2016; Viding & McCrory, 2018). For instance, attenuated response to others’ distress in CP/HCU may facilitate proactive (unprovoked and instrumental) aggression (Fanti, 2018). In contrast, the largely reactive aggression observed in children with CP/LCU group may reflect a defensive reaction to real or perceived threat (Frick et al., 2003). This group show heightened behavioural and neural reactivity to environmental threat stimuli (e.g. fearful faces; Sebastian et al., 2014), and hostile attributional biases, for example interpreting neutral faces as hostile (Dodge & Pettit, 1993; Frick et al., 2003; Dadds et al., 2006). This is likely further compounded by a failure to regulate threat reactivity (Eisenberg et al., 2010; Lickley & Sebastian, 2018).

However, relatively little work has focused on the potential neurocognitive mechanisms underpinning threat reactivity and regulation in this CP/LCU subgroup. Basic neuroscience research on reactive aggression suggests a complex pathway involving both reactivity to stimuli denoting threat, provocation or frustration; and automatic and deliberate regulation mediated by executive processes (e.g. Yu et al., 2014; Hwang et al., 2016; Lickley & Sebastian, 2018). Evidence to date suggests increased *reactivity* to threat in children with CP/LCU, but little is known regarding the interaction between such reactivity and ‘top down’ executive processes. Viding et al. (2012) showed increased amygdala response to facial fear presented preattentively in CP/LCU relative to controls, suggesting hyperreactivity of a ‘bottom-up’ attentional orienting mechanism to threat (Gamer & Büchel, 2009). Sebastian et al. (2014) showed that increased amygdala response when attention was drawn specifically to the salient eye region of fearful faces in children with CP/LCU was associated with increased reaction time (RT) interference on a simple decision-making task. This suggests that such amygdala reactivity may be detrimental for executive task performance. However, it is unclear whether the effect was caused by increased reactivity to stimuli perceived in the same way as in controls, increased exogenous or ‘bottom up’ allocation of attention to emotional aspects of the stimuli, and/or reduced ‘top down’ ability to resolve executive conflict resulting from competing emotional information and cognitive task demands. These possible explanations are not mutually exclusive; however, studies to date have not been designed to tease apart processes occurring at different stages in the information processing stream.

To our knowledge, only one prior study has examined emotion-cognition interactions in CP, taking levels of CU traits into account. Hwang et al. (2016) used an affective Stroop task to assess neural responses to task-irrelevant emotional pictures in young people with CP/LCU and CP/HCU. Participants viewed positive, negative and neutral IAPS images, interspersed with either congruent number Stroop trials (e.g. deciding three numbers are present when all three numerals displayed are ‘3 s’) or incongruent trials (e.g. deciding three numbers are present when three ‘2 s’ are displayed). Across congruency levels, CP/HCU showed reduced amygdala and ventromedial prefrontal cortex (vmPFC) response to negative stimuli compared with healthy youth and CP/LCU. Youth with CP/LCU showed decreased connectivity between amygdala and inferior frontal gyrus in response to emotion in general, potentially suggesting deficient emotion regulation. However, no group differences were seen in the crucial interaction between emotion and compatibility. While this could indicate a genuine null effect, task-specific explanations may also have contributed. For example, the sequential nature of the task design (picture, task, picture) may not drive maximal conflict between emotion and congruency domains (Sebastian et al., 2017). Additionally, IAPS stimuli vary considerably in visual and interpretative properties, which may have increased noise in the emotion contrast. Finally, the use of social stimuli (such as faces) may be more effective, given extensive research suggesting behavioural and neural hypo-reactivity to facial emotions such as fear in CP/HCU and hyper-reactivity in CP/LCU (e.g. Jones et al., 2009; Marsh et al., 2008; Viding et al., 2012; Lozier et al., 2014; Sebastian et al., 2014).

**Fig. 1:**
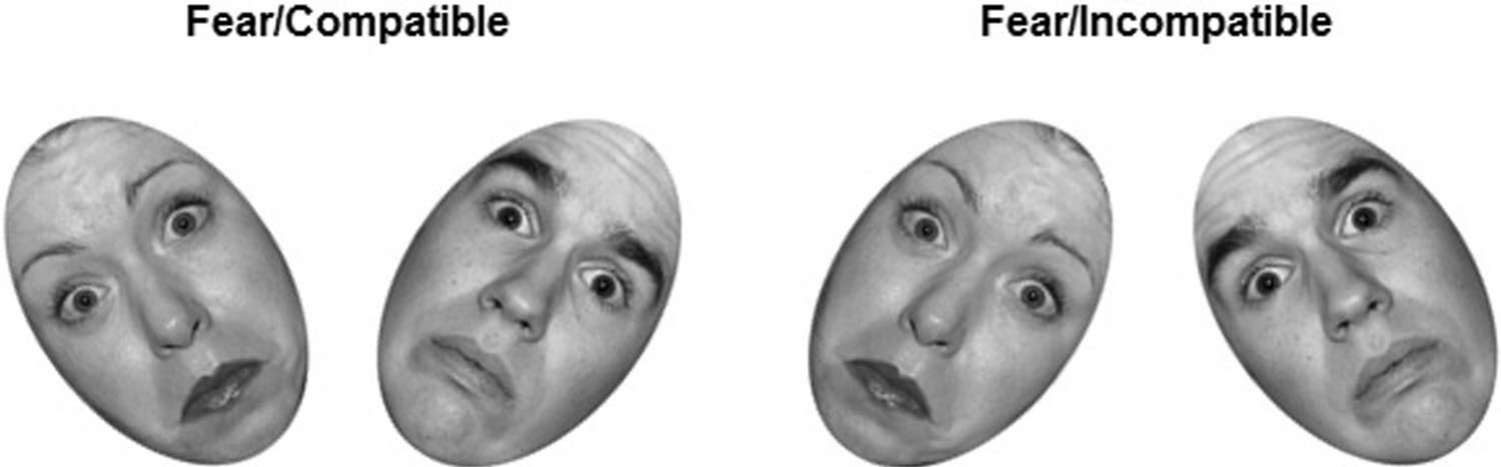
Experimental task stimuli. Each stimulus consisted of two faces; one male, one female. Participants were instructed to identify the face of the target gender (counterbalanced across participants) and indicate with a button press whether it was tilted to the left or right. Facial identities are those for which permission is given to publish from the NimStim, and differed from the identities used in the study

The present study examined emotion-cognition interactions in CP. As discussed, studies to date have not teased apart group differences in exogenous attention to threat vs. cognitive conflict resolution. In a previous study in typical adult males (Sebastian et al., 2017), we developed an emotional face Simon task, which used stimulus–response compatibility vs. incompatibility to vary cognitive load (see Fig. 1) in either the presence or absence of task-irrelevant emotion. The task was designed to match perceptual processing of the emotional information across compatibility conditions, so as to control as far as possible for an exogenous attention-based explanation. Our previous study found decreased amygdala response to fear on incompatible (high load) relative to compatible (low load) trials. No differences were seen for calm faces. This was paralleled by increased RT interference for fear/compatible trials relative to calm, but no RT difference between emotions on incompatible trials. When such a pattern of results is seen in a perceptual load task (in which exogenous attention is manipulated as opposed to controlled, e.g. Pessoa et al., 2002), findings are typically interpreted in line with perceptual load theory (e.g. Lavie, 2005), i.e. on high load trials, attentional processing capacity is focused on the central task, leaving little spare capacity to be captured by task-irrelevant emotional stimuli, which are typically spatially segregated from the relevant task stimulus. However, since perceptual properties were matched across load conditions (participants had to scan emotional faces in the same way for both conditions in order to perform the task), a perceptual load explanation seemed unlikely. We conducted a psychophysiological interaction (PPI) analysis which suggested that the pattern of results seen in the amygdala was driven in part by connectivity between amygdala and middle frontal gyrus, a region previously implicated in emotion regulation (e.g. Blair et al., 2007; Kohn et al., 2014).

We conducted further behavioural work (Ahmed & Sebastian, 2020) to understand why effects appeared to mimic a perceptual load effect (emotion is perceived to a lesser extent under high load) even though task design required that emotion be perceived equally on both compatible and incompatible trials. Tasks where *cognitive* load and emotion interact typically result in *increased* RT interference and (more variably) amygdala response on the crucial high load/emotion condition, not the reverse (Cromheeke & Mueller, 2013). Specifically, we examined stimulus predictability by varying whether compatibility and/or emotion trials were presented in blocks or were randomised. We found that our original interaction effect only replicated when both emotion and compatibility were blocked (and therefore predictable) as in our original study; not when either emotion or compatibility were randomised. Looking across both our previous studies, we concluded that high stimulus predictability may enable the engagement of anticipatory top-down executive control mechanisms for resolving emotional vs. cognitive conflict (as found by Etkin et al., 2006).

We used this task with fMRI to compare children with CP/LCU, CP/HCU and typically developing (TD) controls. We predicted that controls would show the same interaction between emotion and cognitive conflict in the amygdala and RT data as seen in adults (Sebastian et al., 2017; Ahmed & Sebastian, 2020). While it is likely that overall task effects are mediated by cortical-subcortical interactions, we focused our hypotheses on the amygdala in order to reduce the possibility of false positive results. This was the most conclusive result in our previous fMRI study, and is a region where atypical responses have been well-characterised in both CP/LCU and CP/HCU. We further predicted that children with CP/LCU would show dysregulated interactions between emotion and cognitive conflict in amygdala relative to typically developing controls. There were several possibilities as to the form this dysregulated interaction might take: a) CP/LCU could show uniformly increased response to fear across conflict conditions reflecting generalised threat hyper-reactivity and poor modulation of the fear response by cognitive conflict; b) CP/LCU could show a reversed interaction effect with greater amygdala response to fear/incompatible, suggesting poor amygdala regulation of fear under high conflict specifically; c) CP/LCU could show a flat profile of amygdala response suggesting poor modulation of amygdala response by both emotion and conflict under concurrent task conditions. For CP/HCU we predicted reduced overall amygdala reactivity to fear relative to controls and CP/LCU. Regarding load effects, one prior study looking at attentional load found reduced amygdala response to fear in CP/HCU specifically under low attentional load relative to controls (White et al., 2012). However, since studies in the cognitive load domain (e.g. Hwang et al., 2016) have not reported such an effect, and because mechanisms underpinning attentional vs. cognitive load are likely distinct (Lavie, 2005), we did not make specific predictions regarding interactions with cognitive conflict for CP/HCU.

## Methods

### Participants and Procedures

We recruited a sample of fifty-eight boys aged 10–16 from a range of community sources in the United Kingdom including: specialist educational establishments for young people exhibiting social, emotional and behavioural difficulties; mainstream secondary schools; and community advertisements.

Screening questionnaires were used to obtain demographic data and a research diagnosis of either conduct problems (CP; assessed using the Child and Adolescent Symptom Inventory-4R Conduct Disorder subscale; Gadow & Sprafkin, 2009) or typically developing (TD) control status. The Inventory of Callous-Unemotional Traits (ICU; Essau et al., 2006) assessed CU traits, with CP/LCU and CP/HCU group assignment based on a median split (CP median ICU score = 42.5).

Measures of conduct disorder (CASI-CD) and callous-unemotional traits (ICU) were scored by taking the highest ratings from either the parent or teacher questionnaire for each item (Piacentini et al., 1992). The cut-off subscores on the CASI-CD for inclusion in the CP group were: parent report: 4 + (ages 10–12) and 3 + (ages 12–16) or teacher report: 3 + (ages 10–12), 4 + (ages 12–14), and 6 + (ages 15–16). Parents and teachers also completed the Strengths and Difficulties Questionnaire (SDQ; Goodman, 1997) as a broad measure of psychopathology. All TD participants scored below the CP group median on CU traits and within the normal range on each subscale of the SDQ, including CD. Participants selected for scanning additionally completed the Wechsler Abbreviated Scale of Intelligence (WASI; Wechsler, 1999), the Alcohol Use Disorders Identification Test (AUDIT, Babor et al., 2001), and the Drugs Use Disorders Identification Tests (DUDIT, Berman et al., 2005) on the day of the scan. A parent/carer completed CASI-4R measures of ADHD, generalised anxiety disorder (GAD) and major depressive episode (MDE). Those with a diagnosed neurological or psychotic disorder, autism spectrum disorder, or a current prescription for psychiatric medication were excluded. These procedures for screening and group assignment followed our previous studies (e.g. Viding et al., 2012; Sebastian et al., 2014).

Six participants were subsequently excluded for: not meeting criteria for either CP or TD groups (n = 2); not meeting task performance criteria (n = 1); early scan termination (n = 1); excessive motion (affecting > 25% of scans; n = 1), and poor inter-subject registration (n = 1), leaving a final sample of N = 52 across three groups (TD control n = 18; CP/LCU n = 17; CP/HCU n = 17) matched on ethnicity, age, socioeconomic status and IQ (Table 1).

**Table 1:** Demographic data and clinical symptoms, presented by group

Characteristics	TD (n = 18)	CP/LCU (n = 17)	CP/HCU (n = 17)	*P*-value^a^	Post hoc*
Age (years)^b^	14 (1.68)	14 (1.62)	14 (1.93)	0.68	
SES^b^	3.07 (1.01)	3.01 (1.19)	3.48 (1.28)	0.44	
Ethnicity (%)^b,c^	13:2:2:1	13:0:3:1	13:2:2:0	0.82	
IQ (two-subtest WASI)^d^	102.83 (11.69)	104.56 (11.34)	97.41 (15.65)	0.26	
CASI conduct disorder^e^	0.56 (0.70)	6.53 (2.29)	14.24 (6.88)	< 0.001	1 < 2 < 3
ICU^e^	24.17 (4.85)	34.35 (6.53)	51.24 (7.16)	< 0.001	1 < 2 < 3
ADHD^f,g^	9.47 (7.47)	17.64 (10.40)	32.54 (12.75)	< 0.001	1/2 < 3
Generalized anxiety disorder^f^	2.71 (3.07)	5.53 (4.26)	9.67 (5.92)	< 0.001	1/2 < 3
Major depressive episode^f^	2.61 (1.09)	4.80 (3.62)	8.19 (6.80)	0.002	1 < 3
SDQ^e^					
Conduct problems^h^	1.17 (1.47)	4.29 (2.37)	9.59 (5.22)	< 0.001	1 < 2 < 3
Hyperactivity	2.94 (1.80)	6.88 (2.26)	8.71 (1.40)	< 0.001	1 < 2 < 3
Peer problems	1.78 (1.59)	3.76 (2.46)	5.76 (1.95)	< 0.001	1 < 2 < 3
Emotional problems	1.94 (2.29)	3.29 (3.00)	4.53 (2.60)	0.02	1 < 3
Prosocial^i^	10.39 (4.72)	7.65 (1.73)	5.94 (2.30)	0.001	1 > 2/3
Total^h,i^	7.06 (3.78)	18.24 (7.24)	28.59 (7.69)	< 0.001	1 < 2 < 3
Alcohol use and disorders^d^	1.22 (1.99)	1.81 (3.35)	2.71 (4.61)	0.45	
Drug use and disorders^d^	0.17 (0.51)	1.75 (3.92)	3.59 (8.87)	0.2	

*SES* socio-economic status, *WASI* wechsler abbreviated scale of intelligence, *CASI* child and adolescent symptom inventory, *ICU* inventory of callous-unemotional traits, *ADHD* attention deficit hyperactivity disorder, *TD* typically developing, *CP/LCU* conduct problems and low callous unemotional traits, *CP/HCU* conduct problems and high callous-unemotional traits

^*^P < 0.05, Bonferroni corrected

^a^All p-values obtained using ANOVAS except for ethnicity (Chi-square test used)

^b^Measures taken at screening phase, parent/teacher report

^c^White:Black:Mixed: Asian

^d^Child measure/report at scanning session

^e^Measures taken at screening phase, parent report

^f^Measures taken at scanning session, parent report on the CASI-4R

^g^1 participant excluded from CP/HCU group due to missing data on ADHD (CP/HCU n = 16)

^h^1 participant excluded from CP/HCU group due to coding error on SDQ CP subscale (CP/HCU n = 16)

^i^1 participant excluded from TD group due to coding error on SDQ prosocial subscale (TD n = 17)

### Experimental Task

Task procedures followed Sebastian et al. (2017). Participants viewed one male and one female face presented simultaneously (Fig. 1), and had to locate the face corresponding to a target gender (e.g. female). Faces tilted either to the left or to the right, and participants were instructed to make a key press with their right index finger if the target face tilted left, or with their middle finger if the face tilted right, i.e. the response key was spatially compatible with the direction of the tilt. On compatible trials, the target face was located on the same side to which it was tilted (e.g. on the left and tilting left), while on incompatible trials the target face was on the opposite side (e.g. on the right and tilting left). This set up a spatial incompatibility between the required response and its location. Importantly, stimuli must be scanned in the same way on both compatible and incompatible trials to enable gender decision, such that exogenous attention effects are controlled as far as possible.

Emotional Simon task stimuli consisted of two male and two female face identities, each with four different expressions: fear, anger, calm and scrambled (NimStim; Tottenham et al., 2009). Stimuli were greyscale with hair cropped so that participants needed to scan emotion-conveying regions such as the eyes (Adolphs et al., 2005) to complete the gender-decision task. Scrambled stimuli represented a low-level control condition and were created by phase scrambling calm face images (Sadr & Sinha, 2004). Participants indicated their ‘gender’ based on a small pink or blue cross. All faces were rotated along the vertical axis by 35° to the left or right. Paired images of male and female faces with identical expressions were created, half with the female face on the left and half with it on the right. These images were paired such that there were eight possible images (each male with each female) for each expression at each level of stimulus–response compatibility) (64 images in total). Each stimulus array of two faces on a white background measured 606 × 349 pixels and each face oval measured 6 × 4 cm (see Fig. 1).

Stimuli were presented in eight blocks of eight stimuli, one block for each Compatibility (compatible, incompatible) x Face (fear, anger, calm, scrambled) condition. These eight blocks were presented three times, in a different random order each time (192 trials). Participants completed two runs (384 total trials). Randomisation was constrained to ensure all compatible (or incompatible) blocks were not presented sequentially. Within each block, stimuli were randomised with constraints to ensure that all left (or all right) response trials were not presented sequentially. Stimuli were presented for 2000 ms, followed by a fixation cross ISI presented for 500 ms. Each block was therefore 20 s (2500 ms × 8) in duration. A 15 s fixation cross was presented every 4 blocks. Participants completed the task in the MRI scanner using left/right button box responses, and projector system with mirror mounted on the head coil. Prior to scanning, participants completed a short practice task using calm faces not seen in the main experiment, until > 80% accuracy was attained.

### MRI Acquisition

A Siemens Avanto 1.5 T MRI scanner with a 32-channel head coil was used to acquire a 5.5 min 3D T1-weighted structural scan, and two runs of 199 multislice T2*-weighted echo planar volumes with BOLD contrast (~ 10min per run). The EPI sequence was designed to optimise signal detection and reduce dropout in OFC and amygdala (Weiskopf et al., 2006), and used the following acquisition parameters: 35 2 mm slices acquired in an ascending trajectory with a 1 mm gap, TE = 50 ms; TR = 2975 ms; slice tilt = -30° (T > C); flip angle = 90°; field of view = 192 mm; matrix size = 64 × 64.

### Analysis

Behavioural data were analysed in SPSS after removing missed trials and implausible RTs (< 200 ms). Mixed-model ANOVAs were conducted on mean correct RT and percentage error data averaged across runs, with factors Group (TD, CP/LCU, CP/HCU), Emotion (fear, anger, calm, scrambled), and Compatibility (compatible and incompatible).

fMRI analysis was conducted in SPM8. During pre-processing, the first five volumes were discarded to allow for T1 equilibrium, data were realigned, normalised through segmentation of the T1 scan with a voxel size of 2 × 2x2mm, and smoothed with an 8 mm Gaussian filter. Eight regressors of interest were modelled with block duration 20 s, corresponding to each Compatibility x Emotion condition. An additional regressor modelled baseline fixation. These nine regressors were modelled as boxcar functions convolved with a canonical haemodynamic response function. The six realignment parameters were modelled as effects of no interest. Images showing between-scan motion of > 1 mm or 1 degree were individually inspected for distortion. For 9 participants (2 = TD, 1 = CP/LCU, 6 = CP/HCU), extra regressors were included to model images corrupted due to excess motion (less than 10% of each participant’s data). These images were removed and adjacent images were interpolated to prevent distortion of the between-subjects mask. Data were high pass filtered at 128 s to remove low-frequency drifts.

At the first level, main effects of each factor (Compatibility and Emotion) were computed, as well as the interaction of key interest, i.e. fear vs. calm at each level of compatibility, in line with Sebastian et al. (2017). It was decided a priori that neither angry nor scrambled faces would be included in these analyses, since the effect to be replicated in Sebastian et al. (2017) was based on fear vs. calm. Contrasts were then taken up to second-level analysis as t-tests. Amygdala region of interest (ROI) analyses were conducted bilaterally, using 8 mm radius spheres centred on peak right amygdala MNI co-ordinates from Sebastian et al. (2017): right amygdala: 22 -2 -22 and corresponding left amygdala: -22 -2 -22. Results were thresholded at *p* < 0.05 (familywise error-corrected for small volumes (FWE-SVC)), after initial thresholding at *p* < 0.001, uncorrected. Exploratory whole brain analyses for this interaction, as well as for task main effects and group contrasts are reported for completeness in Supplementary Tables 1 and 2, at *p* < 0.05 FWE-cluster level corrected across the whole brain following initial thresholding at *p* < 0.001, uncorrected. Exploratory psychophysiological interaction (PPI) analyses were conducted to explore differences in functional coupling between the amygdala ROI and the rest of the brain in response to fear vs. calm at different levels of compatibility (following Sebastian et al., 2017). The middle frontal gyrus was used as a bilateral ROI, as this region showed functional coupling with the right amygdala on this interaction contrast in our previous study, and is implicated in emotion-cognition interactions. As no significant results were seen in this ROI, further methodological details and exploratory results at an uncorrected threshold of *p* < 0.005, k ≥ *20* are presented in Supplementary Materials.

## Results

### Behavioural Data

#### Reaction Times (RTs)

Results showed a main effect of Emotion: *F*_(3,147)_ = 337.21, *p* < 0.001, η_p_^2^ = 0.87, and followed the pattern scrambled (M = 673 ms, SD = 92) < fear (M = 800 ms, SD = 94) < calm (M = 827 ms, SD = 92) < anger (M = 850 ms, SD = 97; all *p*s < 0.001). There was also a main effect of Compatibility: *F*_(1,49)_ = 206.66, *p* < 0.001, η_p_^2^ = 0.81, driven by significantly faster RTs in compatible (M = 758 ms, SD = 88) relative to incompatible (M = 817 ms, SD = 95) trials. There was no main effect of Group. A marginal interaction was observed between Group and Compatibility, *F*_(2,49)_ = 3.15, *p* = 0.052, η_p_^2^ = 0.11. While all groups had significantly faster RTs in compatible relative to incompatible conditions (*p*s < 0.001), the difference between conditions for CP/HCU was significantly greater than for CP/LCU (*t*_(32)_ = -2.28, *p* = 0.03, Cohen’s d = 0.78). No other significant interactions were observed.

### Mean % Errors

There was a main effect of Emotion (*F*_(3,147)_ = 3.49, *p* = 0.02, η_p_^2^ = 0.07) driven by fewer errors for scrambled faces (M = 2.9%) compared with faces: calm (M = 4.0%, SD = 5.82; *t*_(51)=_2.20, *p* = 0.03, ***d = 0.31***), fear (M = 4.0%, SD = 5.36, *t*_(51)_ = 2.45, *p* = 0.02, ***d = 0.34***), anger (M = 4.4%, SD = 5.08, *t*_(51)=_ = 3.56, *p* = 0.001,There was a main). There was a main effect of Compatibility (*F*_(1,49)_ = 19.32, *p* < 0.001, η_p_^2^ = 0.28) with significantly more errors in the incompatible condition (M = 5.9%, SD = 7.87) than the compatible condition (M = 1.8%, SD = 1.90). There was no main effect of Group.

A marginal interaction was observed between Group and Compatibility (*F*_(2,49)_ = 3.21, *p* = 0.05, η_p_^2^ = 0.12), as well as a three-way interaction between Group, Compatibility and Emotion (*F*_(6,147)_ = 2.66, p = 0.02, η_p_^2^ = 0.10). Both interactions were driven by the CP/HCU group making more errors than the other groups in the incompatible condition (*t*_(50)_ = -2.47, p = 0.17, ***d = 0.73***), particularly for fearful and angry faces. Both were rendered non-significant after accounting for ADHD symptoms, which differed significantly between CP/HCU and the other groups (Table 1) and were hypothesised to influence error rates (Group x Compatibility: *F*_(2,47)_ = 0.48, *p* = 0.62, η_p_^2^ = 0.02; Group x Compatibility x Emotion: *F*_(6,141)_ = 1.83, *p* = 0.10, η_p_^2^ = 0.07).

#### fMRI Data: Amygdala ROI Analyses for the Key Interaction Contrast

### TD Controls

**Fig. 2:**
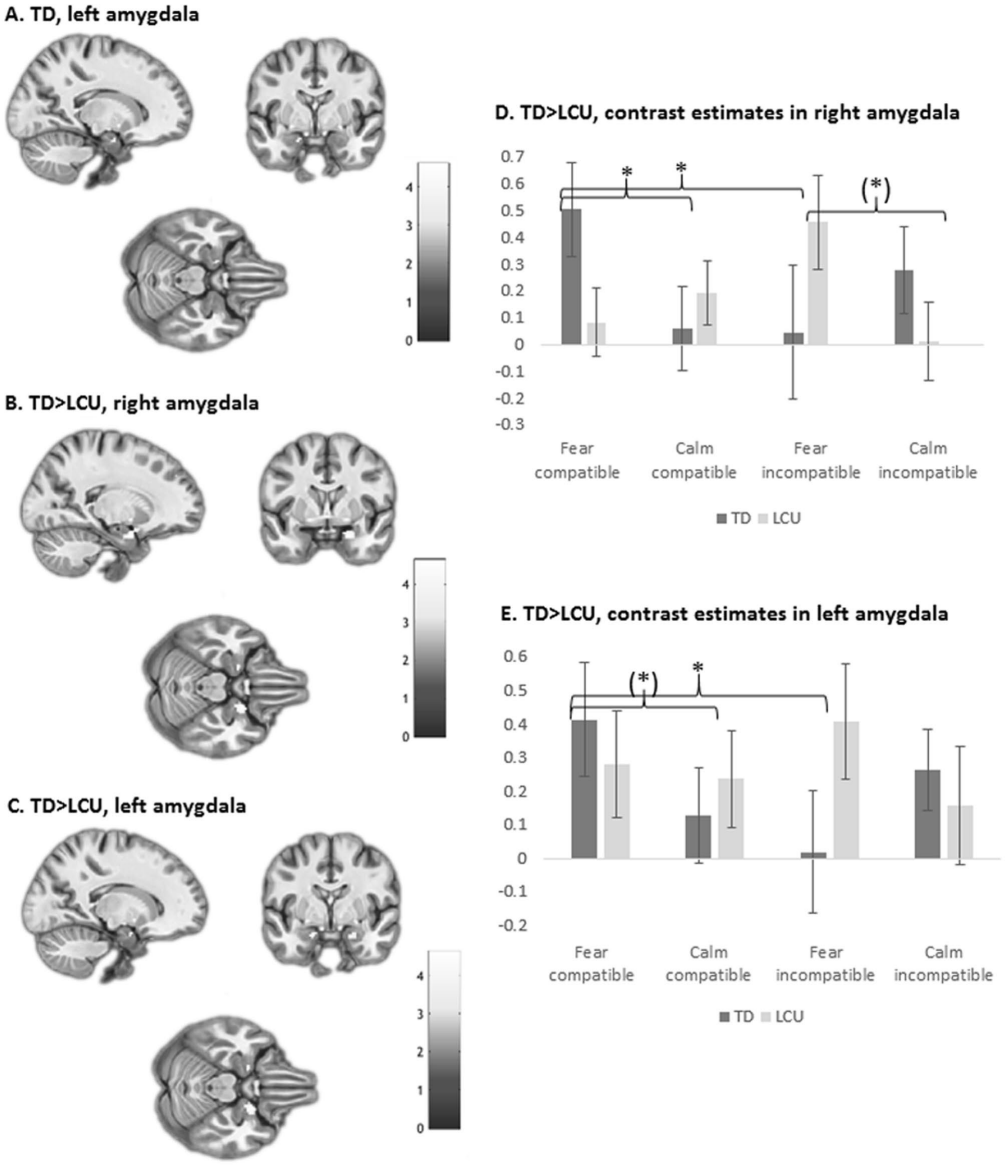
Interaction between fear/calm and compatible/incompatible conditions in the left amygdala for the TD (typically developing) group, and TD relative to LCU (conduct problems with low callous-unemotional traits). **a** Left amygdala activation for the TD group alone (peak voxel: -16 -2 -22). **b** Right amygdala interaction, TD relative to LCU (peak: 20 2 -22). **c** Left amygdala interaction for TD relative to LCU (peak: -16 -2 -22). **d** Contrast estimates for TD vs LCU in the right amygdala. TD: greater response to fear/compatible relative to fear incompatible (*t*_17_ = 2.2, *p* = 0.04), and greater response to fear/compatible than calm/compatible (*t*_17_ = 2.8, *p* = 0.01). LCU: greater response to fear/incompatible relative to fear/compatible (t_16_ = -2.2, *p* = 0.04), and to fear/incompatible compared with calm/incompatible (*t*_16_ = 2.1, *p* = 0.05). **e** Contrasts estimate for TD vs LCU in the left amygdala. TD: greater response to fear/compatible compared to fear/incompatible (*t*_17_ = 2.9, *p* = 0.01), and to fear/compatible relative to calm/compatible (*t*_17_ = 1.9, *p* = 0.07). Results depicted survive small volume correction at *p* < 0.05 (FWE-corrected). Error bars depict standard error of the mean, and colour bars represent t-statistics

We first examined whether attenuated amygdala response to fear on incompatible trials observed in healthy adults (Sebastian et al., 2017) would replicate in the TD control group. With the contrast (fear/compatible > calm/compatible) > (fear/incompatible > calm/incompatible), TD youth showed greater left amygdala response to fear relative to calm in the compatible condition compared to the incompatible condition. Amygdala response was bilateral at uncorrected levels, but only left amygdala survived SVC (peak co-ordinate [x = -16, y = -2, z = -22], *k* = 5, *t* = 3.77, z = 3.52, FWE-SVC *p* = 0.02 (Fig. 2a). Note that this result survived Bonferroni correction across two analyses conducted (right and left amygdala) at *p* < 0.04. Simple effects analysis based on mean contrast estimates across significant voxels extracted using MarsBaR (Brett et al., 2002) showed a very similar profile as in the adult sample. Specifically, the TD group showed increased left amygdala activation for fearful faces in the compatible condition relative to the incompatible condition (*t*_17_ = 2.9, *p* = 0.009, ***d = 0.70***), whereas no significant difference between compatible and incompatible conditions was seen for calm faces (*t*_17_ = -1.2, p = 0.25, ***d = 0.28***). Additionally, the TD group showed increased left amygdala activation for fearful relative to calm faces in the compatible (low conflict) condition which approached significance (*t*_17_ = 1.9, *p* = 0.07, ***d = 0.46***), but this difference was not observed in the incompatible condition (*t*_17_ = -1.3, *p* = 0.20, ***d = 0.31***). This pattern of attenuated amygdala response to fearful faces under high cognitive conflict was not observed across the sample as a whole, or for CP/LCU and CP/HCU groups individually.

### Group Comparisons

We next explored group differences for this interaction contrast within our amygdala ROI. No group differences were found between TD and CP/HCU groups, or between CP/LCU and CP/HCU. However, for CP/LCU vs. TD controls there was a significant group x emotion x compatibility interaction in the amygdala bilaterally: right amygdala peak = [20 2 -22], *k* = 63, *t* = 4.62, *z* = 4.19, FWE-SVC *p* = 0.002; left amygdala peak = [-16 -2 -22], *k* = 11, *t* = 4.21, *z* = 3.87, FWE-SVC *p* = 0.005 (Figs. 2b, c). These differences remained significant after Bonferroni correction for six potential multiple comparisons (three group comparisons (TD vs. LCU, TD vs. HCU, LCU vs. HCU) x left/right amygdala ROIs: right amygdala *p* = 0.012 and left amygdala *p* = 0.03). Results also remained after controlling for anxiety (commonly associated with heightened amygdala response): right amygdala peak = [20 2 -22], *k* = 6, *t* = 3.73, *z* = 3.47, FWE-SVC *p* = 0.034; left amygdala peak = [-18 0 -22], *k* = 6, *t* = 3.65, *z* = 3.41, *p* = 0.034.

Inspection of mean contrast estimates across the right amygdala cluster surviving SVC, extracted using MarsBaR, showed significantly greater amygdala activation in response to fear in the compatible condition compared to incompatible in the TD group, (*t*_17_ = 2.2, *p* = 0.04, ***d = 0.52***), but a greater response on the incompatible condition compared with compatible in the CP/LCU group (t_16_ = -2.2, *p* = 0.04, ***d = 0.54***). Additionally, the TD group showed significantly greater amygdala response to fear/compatible compared with calm/compatible, (*t*_17_ = 2.8, *p* = 0.01, ***d = 0.66***), which was not observed for the CP/LCU group (*t*_16_ = -0.70, *p* = 0.50, ***d = 0.17***; Fig. 2d). Conversely, the CP/LCU group showed a greater response to fear compared with calm in the incompatible condition, (*t*_16_ = 2.1, *p* = 0.05, ***d = 0.51***), which was not observed for the TD group (*t*_17_ = -0.91, *p* = 0.38, ***d = 0.21***). There was no difference between calm/compatible and calm/incompatible for either group.

In the left amygdala, the TD group showed significantly greater activation to fear/compatible compared to fear/incompatible (*t*_17_ = 2.9, *p* = 0.01, ***d = 0.70***) (Fig. 2e), while the CP/LCU group showed no difference between these conditions (*t*_16_ = -0.83, *p* = 0.42, ***d = 0.2***). TD youth also showed a greater response to fear than calm in the compatible condition, which approached significance (*t*_17_ = 1.9, *p* = 0.07, ***d = 0.46***). This was not observed for the CP/LCU group (*t*_16_ = 0.24, *p* = 0.81, ***d = 0.06***). No other significant differences in activation were observed in the left amygdala.

## Discussion

This study investigated the interaction between cognitive conflict and the processing of task-irrelevant emotion in children with conduct problems. We hypothesised that typically developing controls would show a similar profile of amygdala response to typical adults (attenuation of amygdala response to fear relative to calm faces under high (relative to low) conflict) (Sebastian et al., 2017), but that children with CP/LCU and CP/HCU would show different pattern**s**, suggesting dysregulated cognition-emotion interactions. As in typical adults, TD controls showed a significant interaction between Compatibility and Emotion in bilateral amygdala (only left amygdala survived SVC). This was driven by increased amygdala response to fear on compatible trials, but no difference between fear and calm on incompatible trials. Individuals with CP/LCU displayed a significantly different pattern of amygdala response bilaterally relative to TD controls, while children with CP/HCU did not differ from either group. No effects were seen in behavioural data, or in PPI analyses using bilateral amygdala seeds in relation to the hypothesised ROIs, although several findings emerged in the whole brain analyses (see Supplementary Materials).

In right amygdala, children with CP/LCU showed a reversed interaction compared with TD controls, with increased amygdala response on fear/incompatible trials relative to a) fear/compatible and b) calm/incompatible (marginal). On the left, amygdala response did not differentiate between any conditions in CP/LCU. In both adults and TD controls, the most consistent finding has been a strong amygdala response to fear in the compatible condition, which is diminished in the incompatible condition. As perceptual inputs were carefully matched at the design stage, one possible explanation is one of a top-down anticipatory control mechanism engaged preferentially on fear/incompatible blocks to resolve competing emotional and cognitive task demands (e.g. as shown by Etkin et al., 2006, 2011, albeit with effects seen in rostral anterior cingulate cortex rather than amygdala). This may either downregulate amygdala response prospectively, or prevent a costly attentional bias (and attendant amygdala activation) towards affective aspects of the stimuli during the more demanding fear/incompatible trials. However, evidence of such a mechanism in the present study was lacking, since whole brain and PPI results did not reveal activation or connectivity with prefrontal regions associated with cognitive control.

Results in the CP/LCU group suggest reduced flexibility in amygdala modulation by emotion under varying task difficulties (cognitive conflict), either not differentiating across stimulus types, or (in right amygdala) showing increased response on fear/incompatible trials. In other words, CP/LCU may not respond with optimal flexibility to changes in task demands in an emotional context. Increased right amygdala response to fear/incompatible further suggests that this group may preferentially process fear and activate amygdala specifically when cognitive demands are highest, which may not be adaptive in normative environmental contexts. One possibility is that children with CP/LCU may be able to manage competing emotion adequately in the low conflict (compatible) condition, leading to an attenuated response (i.e. no difference relative to calm), but that this mechanism is overwhelmed at higher loads. In support of this explanation, Hwang et al. (2016) found reduced connectivity between amygdala and inferior frontal gyrus on an affective Stroop task, suggestive of poor emotion regulation in individuals with CP/LCU (although this effect did not differentiate conflict conditions). A related explanation may be that the demanding fear/incompatible condition led to negative affect, driving increased amygdala response in line with attentional control theory (Eysenck & Derakshan, 2011). However, such an explanation is typically associated with high anxiety, while covariate analyses suggest that anxiety did not drive results.

Overall, our interpretation of the reversed interaction effect in right amygdala is made with caution, since this pattern was not specifically predicted and was seen only unilaterally. We also saw no group differences in behavioural data, whereas if increased amygdala response on the fear/incompatible condition had maladaptive functional consequences in CP/LCU, we might expect increased RT interference. This lack of a behavioural effect may be driven by increased noise in the current developmental/CP sample relative to previous studies in adults (e.g. mean RT SD across conditions was 88 ms in typical adults in Sebastian et al., 2017, but was 104 ms in TD controls in the present study). It is also possible that the task was not difficult enough to drive group differences (overall mean error rates < 5%).

We also ran PPI analyses to clarify amygdala connectivity profiles (Supplementary Materials). In adults we had previously shown increased coupling between amygdala and middle frontal gyrus specifically during fear/compatible relative to fear/incompatible (accounting for calm), supporting our interpretation that the interaction was driven by top-down modulation. However, amygdala connectivity in this region did not differentiate between groups in the present study, making it difficult to conclusively show that a deficiency in top-down modulation accounts for the pattern of amygdala response seen in CP/LCU (although this interpretation is suggested based on our task design and our prior work using this task).

To our knowledge these data represent one of the first investigations of the neural bases of emotion-cognition interactions in children with conduct problems; a topic with potential translational implications for processes such as emotion regulation (Eisenberg et al., 2010; Schoorl et al., 2016), and threat-reactivity (Frick et al., 2003) in cognitively demanding situations. This is also one of only a handful of studies to characterise potential neurocognitive deficits in children with CP/LCU (Viding et al., 2012; Sebastian et al., 2014; White et al., 2016; Hwang et al., 2016). It adds to an emerging picture of a group of young people engaging in harmful behaviour, underpinned by atypical affective processing at multiple levels, from early pre-attentional orienting (Viding et al., 2012), to emotion-cognition interactions (Hwang et al., 2016), to dysregulated emotion in everyday life (Eisenberg et al., 2010; Cavanagh et al., 2017).

Future studies should clarify which aspects of the CP/LCU profile are most strongly associated with atypical emotion-cognition interactions. For example, this group typically display high irritability (Stringaris & Goodman, 2009), which contributes to dysregulated amygdala-medial PFC connectivity (Stoddard et al., 2017) and cuts across commonly comorbid externalising and internalising diagnoses in youth (Vidal-Ribas et al., 2016). Aggressive behaviour in CP/LCU is also strongly associated with environmental factors such as early maltreatment and harsh parenting (Shields & Cicchetti, 1998; Pollak, 2015; Richey et al., 2016), which have in turn have been linked to a hypervigilant processing style (Pollak et al., 2000; McCrory & Viding, 2015), amygdala hyperreactivity to threat cues (McCrory et al., 2011; Dannlowski et al., 2012), and poor downregulation of aggressive responding (Shackman and Pollak, 2014). However, the extent to which maltreatment-related processes underpin observations in CP/LCU samples remains underexplored.

Finally, results in our CP/HCU group indicated no difference relative to TD controls or CP/LCU. We might have predicted that CP/HCU would show reduced amygdala response to fear relative to TD controls, in line with previous studies using fearful face stimuli (Marsh et al., 2008; Jones et al., 2009; Lozier et al., 2014). However, at least one prior study (Sebastian et al., 2014) has found no difference between CP/HCU and TD controls in amygdala response to fear when participants perform a concurrent cognitive task. Moreover, Hwang et al. (2016) found reduced responses to affective picture stimuli in both amygdala *and* ventromedial prefrontal cortex (vmPFC) in CP/HCU (albeit no interaction with congruency condition), in line with a broader role for a deficit in amygdala-PFC circuitry in this group (Blair, 2007; Marsh et al., 2011; White et al., 2012). The present study found no main effects or interactions involving vmPFC or other PFC subdivisions: further work should elucidate the profile of responding on tasks requiring emotion-cognition interactions in CP/HCU across this broader network tapping affective reactivity and evaluation.

In terms of more general limitations with our sample, participants were male, meaning results cannot be generalised to females with CP. Data were also cross-sectional and so cannot speak to the development of emotion-cognition interactions over time in individuals with CP. Finally, using two groups of children with CP meant that our sample size was reduced, relative to including all children with CP within the same category. Ample evidence supports the use of callous-unemotional traits as a specifier that can reveal very distinct patterns of neurocognitive vulnerability (Frick et al., 2014; Viding et al., 2012) and prior studies indicate that CP and CU can exert suppressor effects in terms of their association with a third variable (e.g. Sebastian et al., 2012; Lockwood et al., 2013). Complex multivariate associations that result from the heterogeneous nature of CP in children, have led to recommendations of sub-group focused analyses, where children with CP are not treated as a single group (Frick et al., 2014).

Nonetheless, although our sample size is in line with or larger than comparable studies from our research group and others (e.g. White et al., 2016; Viding et al., 2012), the findings would benefit from replication with larger groups of TD, CP/HCU and CP/LCU children. A post-hoc power analysis conducted on the group x emotion x compatibility interaction effects seen in amygdala found that power achieved was 87% and 49% for right and left amygdala, respectively (two-tailed, α = 0.05), suggesting that, for left amygdala at least, a larger sample would be required if attempting to replicate the effect. A larger sample would also enable better characterisation of the task in terms of a wider set of ROIs, including close examination of cortical involvement and cortical-subcortical connectivity, which is presumed to mediate the key interaction in the amygdala. However, we would suggest that these smaller and partially exploratory studies are a necessary and valuable stage in the research process, providing the rationale for further confirmatory investigations in larger samples.

In sum, findings demonstrate atypical processing of facial fear under varying cognitive conflict in children with conduct problems and low levels of callous-unemotional traits. This suggests a potentially maladaptive processing style that may contribute to a reduced ability to adapt flexibly to cognitive demands in the presence of competing task-irrelevant emotional stimuli. Somewhat surprisingly, children with conduct problems and high levels of callous-unemotional traits did not differ significantly from TD controls. Further work with larger samples is needed to interrogate cognition-emotion interactions in this group. Overall, findings illustrate the utility of subgrouping young people with conduct problems based on callous-unemotional traits, and highlight a potential neural mechanism that may contribute to reactive aggressive behaviour and poor emotional control in CP/LCU.

## Supplementary Information

Below is the link to the electronic supplementary material.

Supplementary file1 (DOCX 17 KB)

Supplementary file2 (XLSX 14 KB)

Supplementary file3 (XLSX 10 KB)
