# Supplementary material for: Modulation of Amygdala Response by Cognitive Conflict in Adolescents with Conduct Problems and Varying Levels of CU Traits
Source: Res Child Adolesc Psychopathol. 2021 Mar 16;49(8):1043–54. doi: 10.1007/s10802-021-00787-z (PMC8222043; doi:10.1007/s10802-021-00787-z)
Supplement: Supplementary file 1 — Supplementary file1 (DOCX 17 KB) [file 10802_2021_787_MOESM1_ESM.docx]

**Supplementary Materials for ‘Modulation of amygdala response by cognitive conflict in adolescents with conduct problems and varying levels of callous-unemotional traits’**

**Supplementary Results**

**Psychophysiological interactions (PPIs)**

We conducted PPI analyses to understand the connectivity profile of the amygdala during the task: in particular, whether differential connectivity with prefrontal cortex may explain group differences in amygdala response between CP/LCU and TD controls. We conducted two separate PPI analyses using peak coordinates from second level group comparisons in the left and right amygdala, respectively.

For each participant, neural response across the time course was extracted from a volume of interest defined as an 8mm radius sphere around the peak amygdala co-ordinates from the above interaction for TD relative to *CP/*LCU: right amygdala (20 2 -22), and for TD only: left amygdala (-16 -2 -22). This activity comprised the physiological variable, while the interaction contrast comparing fear vs calm at high vs low compatibility comprised the psychological variable. The interaction between these factors (controlling for the main effects of each), shows regions in which functional coupling in the right and left amygdalae, respectively, varies across conditions. In order to examine functional coupling under the conditions of most theoretical interest, we masked results with an additional PPI analysis on the simple effect of ‘fear/compatible vs fear/incompatible’, using the same left and right amygdala seeds (following Sebastian et al., 2017). We focused on a bilateral ROI in the middle frontal gyrus (MFG: right peak=28 50 -10; left peak=-28 50 -10), as this region showed functional coupling with the right amygdala on this interaction contrast in our previous study and is implicated in emotion-cognition interactions. We used an exploratory threshold of p<.005, uncorrected k *≥ 20.*

*Left amygdala*

There was no evidence of functional coupling between the MFG and the left amygdala in ROI analyses for the TD or CP/LCU groups individually, and no differences emerged between groups. In whole brain analyses, no clusters showed significant functional coupling with the left amygdala for the TD group alone. For CP/LCU>TD, clusters survived in the cerebellum (peak=-12 -36 -26, k = 11, t = 3.20, z = 3.04) and right inferior frontal gyrus (peak=28 30 -14, k = 12, t = 3.10, z = 2.95). However, these clusters did not survive masking with the simple effect (fear/compatible > fear/incompatible). No clusters emerged in which functional coupling with the left amygdala was greater for TD compared with CP/LCU.

*Right amygdala*

No functional coupling between right amygdala and MFG was found in either group, and no differences between groups emerged. In exploratory whole brain analyses, the CP/LCU group showed greater functional coupling between the right amygdala and the left temporal pole (peak=-20 4 -20, k = 45, t = 4.20, z = 3.86) and left hippocampus (peak=-18 -18 -18, k = 45, t = 3.45, z = 3.25) than TD controls. The cluster in left hippocampus survived masking with the simple effect (fear/compatible>fear/incompatible). No clusters emerged in which TD showed greater functional coupling with the right amygdala compared with CP/LCU.
